# Chitosan-Based Adsorbents: A Versatile Platform for the Removal of Arsenate and Copper Ions from Water

**DOI:** 10.3390/nano16080458

**Published:** 2026-04-13

**Authors:** Lingli Min, Shuhua Wang, Yuling Li, Yiting Lin, Yulang Chi

**Affiliations:** 1 College of Resources and Environmental Science, Quanzhou Normal University, Quanzhou 362000, China; llmin@qztc.edu.cn (L.M.); shwang@qztc.edu.cn (S.W.); 19837675203@163.com (Y.L.); 18350601790@163.com (Y.L.); 2 College of Oceanology and Food Science, Quanzhou Normal University, Quanzhou 362000, China

**Keywords:** biosorbent, anionic and cationic pollutants, adsorption mechanisms, water purification

## Abstract

Chitosan, owing to its abundant amino and hydroxyl functional groups, serves as an effective biosorbent for the removal of toxic metal(loid) ions from water. This review summarizes recent advances in chitosan-based adsorbents specifically for arsenate (As(V)) and copper ions (Cu(II)), with an emphasis on adsorption mechanisms and electrospun nanofiber technologies. A conceptual “charge adaptation–structure synergy” model is proposed to elucidate the distinct adsorption behaviors of chitosan toward anionic and cationic substances: under acidic conditions, As(V) adsorption is dominated by electrostatic attraction to protonated amino groups, whereas at pH values near or above the pKa, Cu(II) removal proceeds via synergistic chelation involving deprotonated amino and hydroxyl groups. Competitive and synergistic interactions in binary systems, particularly between As(V) and coexisting anions such as phosphate, are also discussed. Notably, the kinetic advantages of electrospun chitosan nanofibers are highlighted, with equilibrium times shortened from several hours to approximately 0.5–2.6 h. Key challenges and future research directions are further discussed, including scalable manufacturing and the treatment of complex wastewater matrices.

## 1. Introduction

Water bodies contaminated with toxic metal(loid) ions remain a significant environmental issue globally [1,2]. Specifically, with the expansion of industrial activities, water contamination has evolved from systems with a single metal(loid) pollutant to those involving multiple metal(loid) contaminants. Among these, As(V) and Cu(II) are of particular concern due to their widespread presence in industrial effluents and mining wastewater, as well as their distinct chemical behaviors and synergistic hazardous effects in co-contaminated areas [3,4]. The International Agency for Research on Cancer classifies arsenic as a Group 1 carcinogen. Its toxicity and mobility are highly species-dependent, and prolonged exposure can result in serious health conditions, including skin cancers and damage to the bladder, liver, and heart [5,6].

Copper, although an essential trace element for human health, becomes a significant environmental pollutant at elevated concentrations [7,8]. In aquatic ecosystems, free copper ions are highly toxic to fish, inducing oxidative stress, gill damage, and bioaccumulation [9]. For humans, acute exposure to Cu(II) typically causes gastrointestinal irritation, whereas chronic overexposure may lead to hepatotoxicity and nephrotoxicity [10].

In China, Hunan, Jiangxi, and Yunnan face considerable arsenic and copper pollution challenges, particularly in mining-affected areas [11,12,13]. In the Shuikoushan mining area of Hunan Province, weathering and leaching of tailings have been identified as the primary contributors to Cu(II) contamination in soils, whereas smelting emissions represent the main source of arsenic [12]. Similarly, studies conducted around the Dexing mining area in Jiangxi Province—one of China’s largest copper production bases—have revealed severe soil contamination by both copper and arsenic, with mining activities considered the principal source of both elements [11]. These make advanced purification an urgent need.

Common techniques for treating combined metal(loid) pollution include chemical precipitation, ion exchange, and membrane separation [14]. However, these methods often exhibit limitations in efficiency due to issues such as sludge generation, high operational costs, and substantial energy consumption [15,16]. These challenges have heightened the demand for novel bioadsorbents that are efficient, selective, environmentally sustainable, and cost-effective. In this context, chitosan has garnered considerable interest owing to its structural versatility and environmental benefits. Chitosan can be derived from a wide range of natural sources, from conventional crustacean shells to emerging alternatives such as insects and fungal mycelia, with extraction yields and physicochemical properties varying significantly depending on the biological origin [17,18].

**Figure 1:**
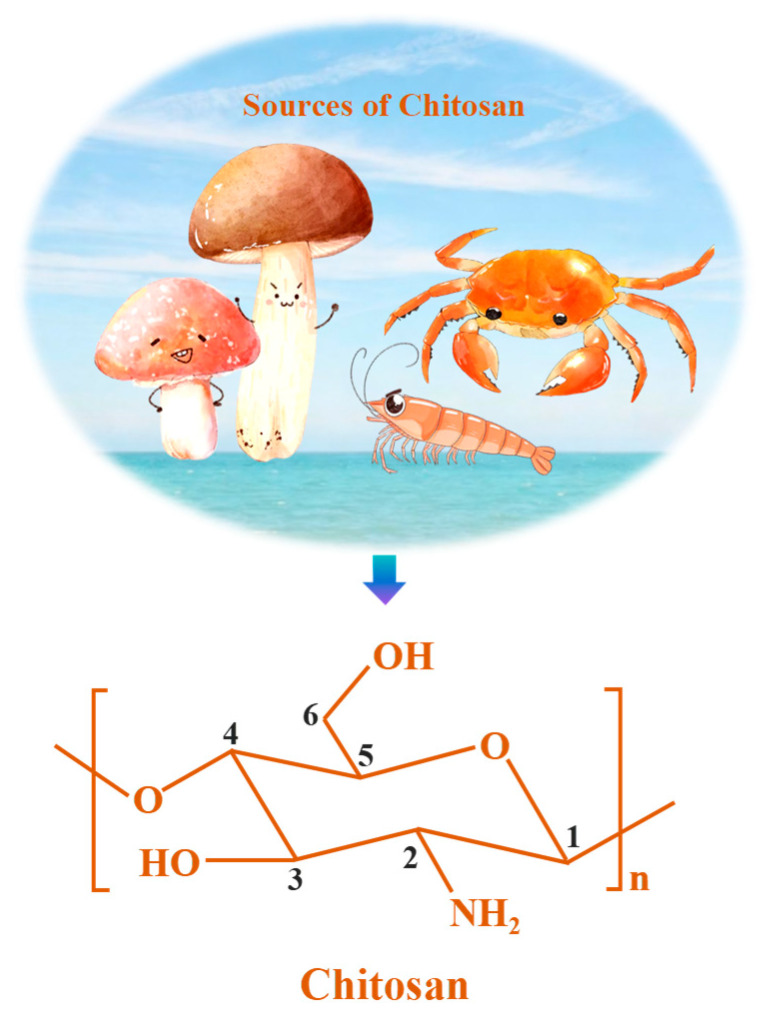
The sources and molecular configuration of chitosan. Numbers 1–6 denote the carbon atoms of the pyranose ring (C1–C6), with C2 carrying the characteristic amino group.

Regardless of the source, chitosan molecular chains are rich in amino (–NH_2_) and hydroxyl (–OH) groups (Figure 1), which facilitate the capture of toxic metal(loid) ions through mechanisms including electrostatic attraction, ion exchange, and surface complexation [19,20]. This structural configuration enables chitosan to adsorb pollutants with different charges—such as anionic arsenic species and cationic copper ions—via distinct yet complementary mechanisms, making it a highly promising material for the remediation of combined contamination [21]. Nevertheless, the majority of current research remains focused on single-ion systems [22].

In contrast to single-ion studies, understanding the competitive and synergistic interactions between coexisting ions is essential for practical applications. Accordingly, this review examines the progress in the application of chitosan-based biosorbents for the removal of As(V) and Cu(II) from aqueous solutions. It proposes a conceptual “charge adaptation–structure synergy” model, which, unlike conventional frameworks that treat electrostatic attraction and chelation as separate phenomena, shows how these two mechanisms operate in a complementary yet context-dependent manner to govern the adsorption of anionic and cationic pollutants onto chitosan. The interaction processes, kinetic characteristics of As(V) and Cu(II) adsorption onto chitosan-based adsorbents, and practical strategies are compared and synthesized. The objective is to offer new insights for the targeted design of high-performance chitosan-based adsorbents and for the optimization of remediation approaches in complex aquatic environments.

## 2. Overview of Chitosan

**Figure 2:**
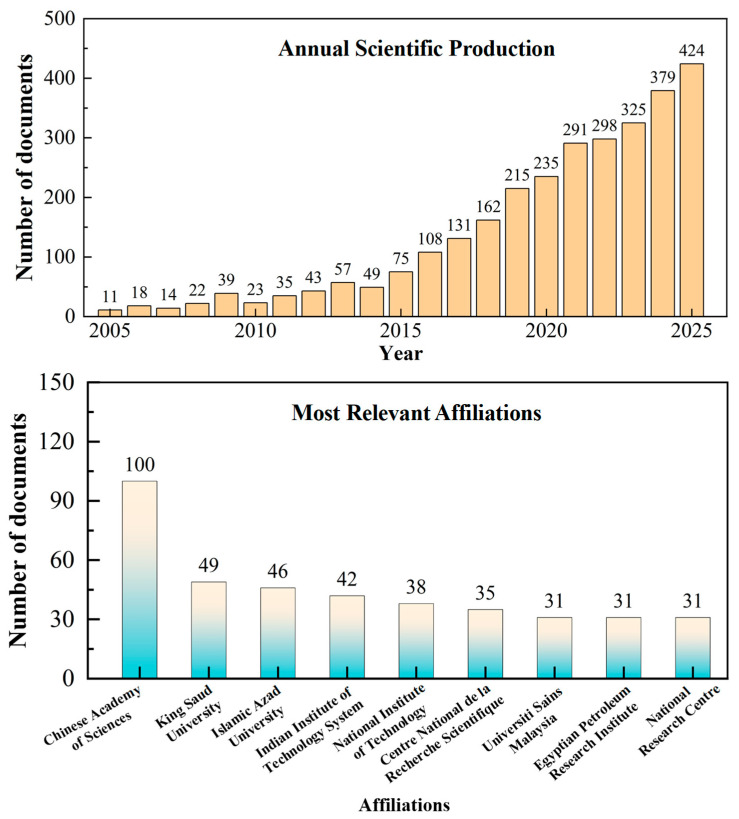
Annual publications from 2005 to December 2025. Top affiliations according to the number of papers published. Data source: Web of Science Core Collection; searched by topic (chitosan, water treatment, adsorption) on 7 March 2026.

Chitosan, the most abundant natural alkaline polysaccharide after cellulose, is primarily derived from the discarded exoskeletons of crustaceans such as shrimp and crabs [23]. These shells typically contain about 20–30% chitin by dry mass, alongside proteins and calcium carbonate [24,25,26]. The conversion of chitin into chitosan via deacetylation exemplifies a “waste-to-resource” strategy aligned with circular economy principles, underscoring the sustainability of chitosan as a raw material. Our examination of papers in the Web of Science Core Collection from 2005 to 2025 shows a significant increase in research on chitosan-based adsorption for water treatment. As illustrated in Figure 2, which was created from our statistical results, the annual number of publications increased substantially from 11 in 2005 to 424 in 2025, demonstrating rapidly expanding research interest in this topic. In terms of contributing institutions, the Chinese Academy of Sciences and King Saud University emerged as the most prolific, showing their leadership roles in promoting chitosan-based adsorption technology.

The growing research interest in chitosan-based adsorption technologies stems largely from chitosan’s unique structural characteristics, which provide it distinct advantages over conventional synthetic adsorbents for water treatment applications. First, its biodegradability offers a notable environmental benefit: natural enzymes such as chitinase and lysozyme can degrade chitosan into non-toxic oligosaccharides and glucosamines [27,28,29], thereby mitigating secondary pollution associated with the disposal of exhausted adsorbents. Second, chitosan contains an abundance of reactive functional groups. The amino groups at the C2 position and the hydroxyl groups at the C3 and C6 positions enable diverse modification strategies—including protonation, coordination, and graft copolymerization—while also serving as potential chelation sites for metal sequestration [30]. Third, the safety and biocompatibility of chitosan are well established. Chitosan has received Generally Recognized as Safe (GRAS) status from the U.S. Food and Drug Administration (FDA) for use as a food ingredient (FDA GRAS Notice No. 997), and multiple chitosan-based wound dressings have been cleared by the FDA through the 510(k) pathway. These regulatory approvals support its potential for safe use in drinking water treatment. Beyond water treatment, chitosan has also been explored in energy storage applications, such as supercapacitors, lithium-ion batteries, and fuel cells [31,32].

However, chitosan-based adsorbents have several inherent limitations, such as restricted adsorption capacity, prolonged equilibrium times, and the potential leaching of adsorbent particles, which restrict their widespread practical application [5]. Recent efforts to address these challenges have focused on two primary strategies: composite modification—through the incorporation of inorganic nanoparticles or organic polymers—and morphological control, exemplified by the fabrication of gel microspheres or nanofibers [33].

Of these, electrospun chitosan nanofiber adsorbents have emerged as a particularly promising approach, especially in improving adsorption kinetics [34,35,36]. Ion diffusion paths are significantly reduced, and the time needed to reach equilibrium is accelerated by their high specific surface area, linked pore network, and variable surface chemistry [37]. For example, our research has shown that electrospun chitosan-based nanofiber membranes can remove arsenic from contaminated water quickly and effectively by lowering the equilibrium time for arsenic adsorption from several hours, which is typical for many bio-adsorbents, to roughly 0.5–2.6 h [34,35,36]. As indicated in Table 1, the release of several review publications in 2025 concentrating on electrospun nanofibers for environmental applications demonstrates the growing interest in electrospun nanofibrous membranes for water treatment. The recent increase in scholarly interest in this material platform demonstrates how promising it is.

Fundamentally, the adsorption performance of chitosan is governed by the pH-dependent interplay of its functional groups. As a cationic polyelectrolyte, chitosan exhibits a charge density that varies with solution pH. The dissociation constant (pKa) of the amino groups is approximately 6.3–6.5 [44,45]. In acidic conditions (pH 2–6), the amino groups undergo protonation (–NH_3_^+^), conferring a net positive charge on the polymer surface. This protonated state facilitates the efficient electrostatic capture of anionic contaminants, such as As(V) oxyanions [46]. Conversely, in near-neutral to slightly alkaline conditions (pH 6–8), the amino groups become deprotonated and can coordinate with adjacent hydroxyl groups to form stable chelation sites, enabling the selective binding of cationic metals [47].

## 3. How Chitosan-Based Adsorbents Remove As(V) and Cu(II)

The adsorption properties of chitosan regarding As(V) and Cu(II) show significant sensitivity to pH and a notable structural relationship. The protonation state of surface functional groups and the chain conformation are the main factors that affect adsorption performance [48]. Research shows that chitosan chains tend to pack tightly together near the isoelectric point because they have a net zero charge. When the pH of the solution is different from the isoelectric point, chain segments expand because of charge repulsion [49,50]. This directly controls how easily target ions can reach active sites.

### 3.1. Mechanisms for the Removal of As(V)

As(V) binds to chitosan and its derivatives in a variety of ways. The key mechanisms are outlined as follows.

(1) Electrostatic attraction among protonated amino groups: As shown in Figure 1, chitosan contains numerous amino and hydroxyl groups throughout its molecular structure. When the pH is low, the amino groups gain protons, which gives the polymer positive charge. Under acidic conditions (pH 3–6), As(V) exists predominantly as negatively charged H_2_AsO_4_^−^ due to its pKa values (pKa_1_ = 2.2, pKa_2_ = 6.9) [51]. Chitosan’s protonated amino groups are the sites where As(V) adhere. This means that the adsorption is mostly an electrostatic interaction between negatively charged As(V) and positively charged amino groups that have been protonated. This process depends a lot on the pH. When the pH is low (pH 2–5), amino groups are heavily protonated, which makes it easier for As(V) to be taken up.
(2) Coordination and surface complexation: Beyond electrostatic attraction, the adsorption of As(V) onto chitosan-based adsorbents can occur through coordination interactions, a behavior governed by the coordination chemistry of the amine groups and the stability constants of the resulting complexes [52]. This effect is particularly pronounced when the adsorbent is functionalized with metal ions such as iron or molybdenum, albeit through distinct mechanisms. For iron (Fe), Fe(III) forms inner-sphere complexes with As(V) via ligand exchange, creating stable bidentate binuclear surface complexes. In chitosan–Fe composite systems, chitosan acts primarily as a supporting matrix that disperses and stabilizes Fe oxides, while the high affinity between Fe–O and As–O bonds drives the adsorption [38]. For molybdenum (Mo), under acidic conditions, molybdate ions (MoO_4_^2−^) pre-adsorbed onto chitosan can form complexes with As(V) [53,54]. These metal-functionalized chitosan composites thus combine the coordination capacity of the metal centers with the structural advantages of the chitosan matrix, enhancing both adsorption capacity and selectivity for As(V).
(3) Special mechanisms encompass redox reactions and competitive adsorption: In intricate composite systems, chemical transformations above simple adsorption have been noted. A 2023 investigation identified a redox mechanism in magnetic iron-based alginate-chitosan beads that absorbed As(V). In anoxic aqueous solutions, the Fe(II) in the material can transform adsorbed As(V) into As(III). Subsequently, As(III) demonstrated patterns of adsorption, desorption, and re-adsorption. In oxygen-enriched air, As(V) donated electrons to Fe(II) and O_2_, leading to the formation of As(III), which can subsequently be re-oxidized to As(V) [55]. This redox transformation is environmentally relevant because As(III) is more mobile and significantly more toxic than As(V) [56]. Therefore, while such redox-active composites may enhance total arsenic removal under certain conditions, careful consideration must be given to the potential release of more toxic As(III) during desorption or under anoxic conditions.
(4) Perspectives from molecular dynamics: In recent years, computational chemistry has offered a detailed understanding of the mechanisms involved in adsorption at the atomic level [57]. The temporal interactions between chitosan and arsenic acid were investigated using computational approaches [58]. The study found that the adsorption process occurs in two distinct stages: first, weak hydrogen bonding takes place, followed by the formation of a double-layer adsorption. The primary layer is formed by the direct interactions between chitosan and arsenic acid molecules. In contrast, the secondary layer develops from the collective aggregation of arsenic acid molecules and their interactions with the primary layer [58]. These findings offer a theoretical basis for understanding multi-layer adsorption behavior and the stability of the adsorption system.

### 3.2. Mechanisms for the Removal of Cu(II)

The adsorption of Cu(II) onto chitosan-based adsorbents entails many pathways, predominantly influenced by the plentiful amino and hydroxyl functional groups present in the chitosan backbone. The fundamental mechanisms can be encapsulated as follows: chelation/complexation, electrostatic attraction, and augmented coordination via added functional groups. Spectroscopic methods like Fourier transform infrared (FTIR) and X-ray photoelectron spectroscopy (XPS) have been crucial in clarifying these interactions at the molecular scale.

(1) Chelation and the development of coordination complexes: The primary process for Cu(II) adsorption on chitosan involves the formation of coordination complexes between Cu(II) and the electron-dense functional groups of chitosan, especially the amine (–NH_2_) groups. Yamani et al. delineated the binding mechanisms of Cu(II) to chitosan specifically [59]. Cu(II) is reported to bind to the amine moiety on the chitosan backbone as a monodentate complex (Type I) and a bidentate complex (Type II), the latter crosslinking two polymer chains [60]. The development of these complexes is contingent upon pH and copper concentration [59]. The Type I complex exists independently under threshold circumstances; however, above pH 5.5 during synthesis and with a copper loading of 0.25 mol Cu^2+^/mol chitosan monomer, both Type I and Type II complexes coexist. This coordination is fundamental to chitosan’s capacity to immobilize Cu(II). Fatinathan et al. examined Schiff base-modified chitosan beads (altered with benzaldehyde) and determined that chemisorption was the rate-limiting step in Cu(II) adsorption [61]. The authors suggested that the electron-dense functional groups on the modified chitosan could establish coordination complexes with Cu^2+^. This study further established that the electron configuration of Cu(II) promotes better interactions with the hard ligands found in chitosan.
(2) Electrostatic attraction: In acidic-to-near-neutral pH settings, the amino groups of chitosan become protonated (–NH_3_^+^), facilitating electrostatic interactions with copper species, especially when copper exists as anionic complexes alongside coexisting ligands [62]. Guzman et al. conducted a comprehensive study on the sorption of Cu(II) by chitosan in the presence of citrate ions [62]. Their findings indicated that, under acidic conditions, Cu(II) uptake occurs primarily through electrostatic interactions between protonated amine groups on chitosan and anionic copper-citrate complexes, mainly Cu(OH)L^2−^ and, to a lesser extent, CuL^−^. The adsorption mechanism shifts from ion exchange to electrostatic attraction depending on the pH and the prevailing copper species. Sorption becomes significant only when the proportion of anionic copper complexes exceeds that of anionic ligands not bound to copper [62]. In a related study, Li et al. investigated SiO_2_/chitosan hybrid aerogels for Cu(II) removal and demonstrated via FTIR and XPS spectral analysis that electrostatic attraction plays a key role in the adsorption mechanism [63].
(3) Cooperation among several mechanisms in composite systems: In intricate composite materials, many mechanisms frequently function concurrently. Wu et al. examined alginate-based beads augmented with polysaccharides for Cu(II) adsorption and determined that the adsorption mechanism encompassed ion exchange, chelation, and electrostatic interaction [64]. Their spectroscopic investigation validated chemical interactions between the beads and Cu(II). A 2024 investigation on Fe-modified magnetic chitosan for the co-adsorption of tetracycline and Cu(II) demonstrated that in binary systems, the presence of Cu(II) and tetracycline significantly enhanced their mutual removal by the formation of ternary complexes (adsorbent-Cu^2+^-TC or adsorbent-TC-Cu^2+^) [65]. This illustrates that Cu(II) can function as a bridging ion between the adsorbent and other contaminants, thus enhancing the mechanistic comprehension beyond just binary adsorption.

In short, the removal of Cu(II) onto chitosan entails many chemical and physical processes, predominantly governed by functional groups. Key processes include the formation of coordination complexes through chelation, especially with amine groups, and electrostatic attraction in specific conditions.

### 3.3. Charge Adaptation-Structure Synergy Model

Chitosan-based adsorbents remove As(V) and Cu(II) through a combination of electrostatic interactions, ion exchange, and surface complexation [66]; however, the dominant mechanisms and their driving forces differ significantly between anionic and cationic species (Table 2). Toward a more comprehensive molecular-level understanding, this review proposes a “charge adaptation–structure synergy” model, which incorporates recent experimental and computational insights into the distinct adsorption behaviors of chitosan toward As(V) and Cu(II).

(1) Charge Adaptation-Dominated Mechanism for As(V)

**Figure 3:**
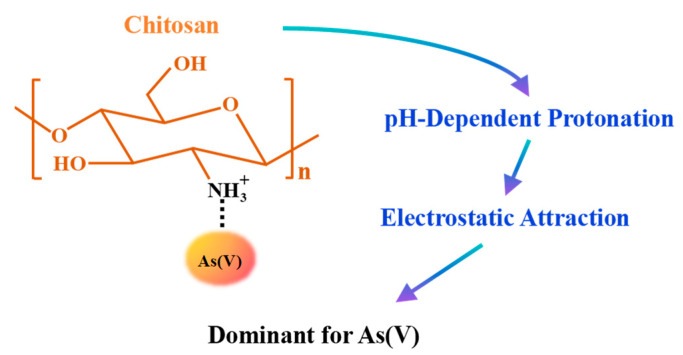
Charge Adaptation-Dominated Mechanism for As(V).

As detailed in Section 3.1, the adsorption of As(V) onto chitosan under acidic conditions is primarily driven by electrostatic attraction between anionic As(V) species and protonated amino groups. This behavior exemplifies “charge adaptation,” whereby chitosan’s functional groups reversibly adjust their protonation state in response to pH (Figure 3), enabling dynamic charge matching with target pollutants.

Quantitatively, electrostatic interactions can account for 70–80% of the total As(V) adsorption capacity under optimal conditions, as supported by surface complexation modeling and zeta potential measurements [46,52]. Moreover, when the solution pH deviates from the isoelectric point of chitosan, the molecular chains adopt a more extended conformation due to interchain electrostatic repulsion, which enlarges internal diffusion channels and facilitates the transport of As(V) anions to buried active sites [48]. Thus, for As(V), the adsorption process is primarily governed by “charge adaptation,” with electrostatic attraction serving as the key driving force.

(2) Structure Synergy-Dominated Mechanism for Cu(II)

**Figure 4:**
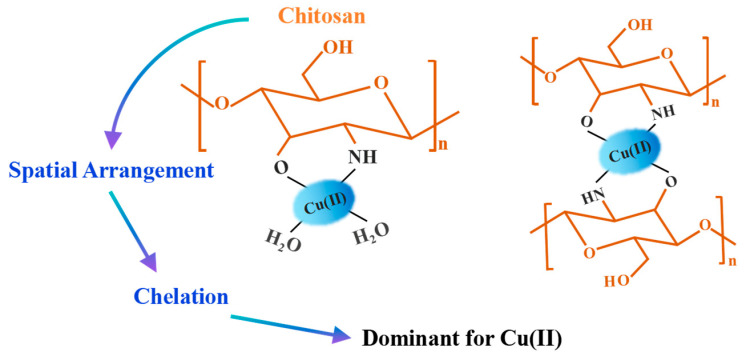
Structure Synergy-Dominated Mechanism for Cu(II).

In contrast, Cu(II) exists as a divalent cation under most environmentally relevant pH conditions. However, at pH values below approximately 6.5, the chitosan surface is positively charged (due to –NH_3_^+^), which would electrostatically repel Cu(II) cations. The effective removal of Cu(II) under near-neutral conditions therefore relies on a different principle: structural synergy (Figure 4).

This concept is based on the unique spatial arrangement of functional groups along the chitosan backbone. Specifically, the amino group at the C2 position and the hydroxyl group at the C3 position are orientated in a 1,3-relationship along the glucosamine ring, providing an ideal span for the formation of a five-membered chelate ring upon simultaneous coordination with a single Cu(II) ion (Figure 4). This bidentate chelation mode is well documented by spectroscopic (FTIR, XPS, EPR) and computational studies [59,60,62]. The formation constant (log K) of the Cu(II)–chitosan complex is typically in the range of 4–5, significantly higher than that of monodentate amine complexes (e.g., log K ≈ 2–3 for Cu(II)–NH_3_), reflecting the entropic and enthalpic advantages of chelation [60]. The chelate effect is thermodynamically driven by a favorable entropy change (ΔS > 0), as the displacement of coordinated water molecules by a single multidentate ligand increases the overall number of free species in solution.

Beyond chelate thermodynamics, structural synergy also encompasses geometric and electronic complementarity. Cu(II) typically adopts a distorted octahedral coordination geometry due to the Jahn–Teller effect; in many chitosan–Cu(II) complexes, the equatorial plane is formed by the nitrogen and oxygen atoms of the chelating ligands, while axial positions are occupied by weakly coordinated water molecules [60,61]. The five-membered chelate ring formed by the C2 amino and C3 hydroxyl groups readily accommodates this geometric preference. Moreover, the amino group acts as a hard Lewis base while the hydroxyl group behaves as an intermediate base; their combination provides electronic complementarity that matches the borderline Lewis acidity of Cu(II) [60], resulting in enhanced complex stability. Consequently, this structural synergy expands the effective pH window for Cu(II) adsorption (from pH~5 to 8) and confers selectivity over competing cations that lack compatible coordination geometries.

Importantly, the “structure synergy” concept emphasizes that molecular architecture—specifically the spatial and electronic complementarity between chitosan’s functional groups and the target ion—is the decisive factor for Cu(II) binding. This mechanism operates in concert with charge adaptation described in the previous section, together enabling chitosan to address both anionic and cationic contaminants through distinct but complementary pathways.

(3) Complementary Roles and Model Summary

Notably, these two mechanisms are not mutually exclusive; under intermediate pH conditions or in composite systems, they may operate concurrently. For instance, the presence of additional functional groups (e.g., carboxylates, metal oxides) can introduce hybrid binding modes that combine electrostatic attraction with chelation.

By explicitly separating the roles of “charge matching” and “structural compatibility,” this model provides a rational foundation for the targeted design of high-performance chitosan-based adsorbents. Charge matching is governed by the pH-dependent protonation equilibrium of the amino groups (pKa ≈ 6.3–6.5), allowing the adsorbent surface to dynamically adjust its net charge in response to solution pH [46,48]. Structural compatibility arises from the 1,3-arrangement of the C2 amino and C3 hydroxyl groups along the glucosamine ring, which provides an ideal spatial configuration for bidentate chelation, while their complementary electronic properties ensure effective coordination with target metal ions [59,60]. To further enhance practical applicability, future materials can be engineered to either enhance charge adaptation (e.g., through quaternization to expand the cationic working pH range) or amplify structural synergy (e.g., by grafting additional chelating ligands to improve selectivity for specific metal ions). This conceptual framework thus bridges molecular-level understanding with practical adsorbent development.

### 3.4. Electrospun Chitosan Materials

The structural synergy concept outlined above highlights the importance of molecular architecture; however, realizing this potential in practical adsorbents also requires optimal macroscopic morphology. Chitosan-based adsorbents have been fabricated into various forms, including hydrogels, beads, membranes, and composite particles, each with distinct advantages in specific applications [33,52]. Among these, electrospun nanofibers have garnered particular attention due to their unique combination of structural features that directly address the key limitations of conventional chitosan adsorbents—namely, slow adsorption kinetics and limited accessibility of active sites [67,68,69]. Given these notable advantages, this section therefore focuses on electrospun chitosan materials, highlighting how their architectural characteristics translate into enhanced adsorption performance, while also discussing the design flexibility that makes them a promising platform for advanced water treatment.

Electrospinning has emerged as a versatile and powerful technique for the direct conversion of chitosan solutions into continuous fibers with diameters spanning the nano- to micro-scale [67]. This process, driven by a high-voltage electric field, facilitates the assembly of these fibers into a three-dimensional non-woven mesh characterized by an exceptionally high specific surface area and interconnected porosity [68]. This unique architecture is pivotal in overcoming the inherent performance limitations of conventional chitosan adsorbents, such as cast films or beads, which often suffer from significant mass transfer resistance and the inaccessibility of active sites buried within their dense matrix [67]. By reducing the characteristic dimensions for diffusion and rendering the functional binding sites readily accessible, the electrospun mat architecture offers a strategic solution to these challenges [69]. Consequently, electrospun chitosan-based materials exhibit improved adsorption kinetics.

**Figure 5:**
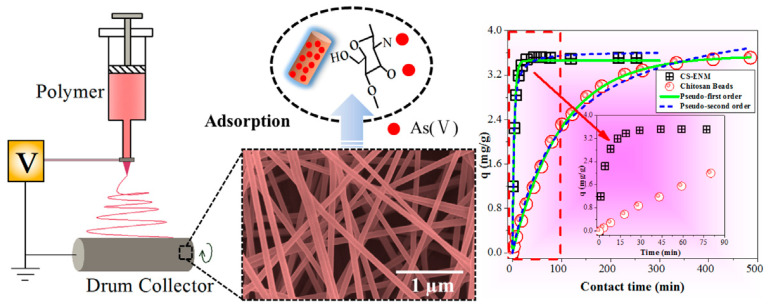
Preparation and characterization of chitosan electrospun membranes (CS-ENM) and comparison of their adsorption kinetics with chitosan beads [34]. (**Left**)—schematic diagram of electrospinning; (**Middle**)—scanning electron microscopy (SEM) image of chitosan nanofiber membrane; (**Right**)—comparison of adsorption kinetics equilibrium time between CS-ENM and chitosan beads.

The improvement in kinetic performance is a direct consequence of the structural attributes imparted by electrospinning. The high porosity and nanoscale diameter of the fibers create short diffusion pathways, allowing target contaminants to reach interior adsorption sites more rapidly [70]. This contrasts with conventional dense adsorbents, where intraparticle diffusion is often the rate-limiting step, leading to longer contact times [71]. The accessibility of functional groups (e.g., amine and hydroxyl groups) on the large surface area of the nanofibers further supports the adsorption process [67,69]. As shown in Figure 5, a quantitative illustration of this kinetic advantage is provided by the adsorption of As(V) [34]. Under the same experimental conditions (initial concentration of 2 mg/L, pH 4.5, 25 °C), electrospun chitosan fibers achieved an adsorption capacity of 3.5 mg/g within 50 min. In comparison, conventional chitosan beads adsorbed only 1.1 mg/g over the same period [34]. Similar rapid adsorption kinetics have been demonstrated for electrospun chitosan-based adsorbents in the removal of Cu(II), Cr(VI), and Cd(II) [72,73,74]. These observations indicate that adsorbent architecture has a substantial influence on mass transfer.

To further illustrate the performance advantages of electrospun architectures, Table 3 compares the Cu(II) adsorption capacity of representative chitosan-based adsorbents reported in the recent literature (2020–present). The data show that electrospun nanofibers generally achieve higher adsorption capacities and faster equilibrium times compared to conventional bead or hydrogel formulations, consistent with their structural features.

Consistent with the kinetic benefits noted above, the time required to reach adsorption equilibrium for As(V) is considerably reduced when using electrospun materials. Traditional chitosan-based adsorbents, such as beads or flakes, typically require equilibrium times ranging from several hours to tens of hours due to their dense structures [81,82]. In contrast, electrospun chitosan membranes have been shown to achieve equilibrium in a mere 0.5 to 2.6 h, a 5- to 10-fold improvement in process efficiency [34,35,36,69].

Beyond their adsorption kinetics, electrospun chitosan materials offer notable flexibility in structural and functional design. The electrospinning process allows for tailoring adsorbent properties by modulating key parameters. Solution properties (such as polymer concentration, viscosity, and solvent system), processing conditions (including applied voltage, solution flow rate, and needle-to-collector distance), and ambient parameters (temperature and humidity) can be adjusted to influence fiber diameter, morphology, and mat architecture [68,83]. For instance, response surface methodology has been employed to optimize these parameters, enabling the fabrication of fibrous networks with average diameters as low as 92 nm [84], thereby increasing the specific surface area available for adsorption. The three-dimensional network structure can also be optimized to reduce diffusion resistance, while fiber porosity and swelling behavior can be tuned to further influence adsorbate-adsorbent interactions [85].

This design flexibility extends to the chemical composition of the fibers. Functional components, such as nanoparticles (e.g., iron oxides for enhanced arsenic affinity), other polymers (e.g., polyethylene oxide to improve spinnability or polyvinyl alcohol for mechanical reinforcement), or active molecular agents, can be incorporated into the spinning dope [35,36]. This one-step fabrication of composite materials allows for the integration of chitosan’s inherent chelation ability with the specific functionalities of additives, producing adsorbents with enhanced capacity, selectivity, or multifunctional properties (e.g., antimicrobial activity) [68]. Collectively, this tunability supports the rational design and targeted optimization of electrospun chitosan membranes for the removal of specific contaminants, making them a promising platform for water purification technologies.

### 3.5. Competitive and Synergistic Adsorption in Binary Systems

In practice, however, practical water bodies often contain multiple toxic metals and coexisting anions, leading to complex competitive or synergistic interactions that can influence adsorption performance—an aspect that remains less explored in studies focused on single-pollutant systems.

#### 3.5.1. Competition Between As(V) and Phosphate

Phosphate (PO_4_^3−^) is a particularly strong competitor for As(V) adsorption due to its similar tetrahedral oxyanion structure and higher abundance in natural waters. Yamani et al. systematically investigated the selectivity of chitosan–copper beads (CCB) for As(V) in the presence of phosphate [59]. They reported that the coordination mode of Cu(II) on chitosan—monodentate (Type I) or bidentate (Type II)—depends on pH and copper loading. Type I complexes (formed at Cu loading <0.25 mol Cu per mol of chitosan monomer) favored phosphate chelation, whereas Type II complexes (formed at higher Cu loading) favored As(V) chelation. Binary separation factors (α_12_) confirmed that the Type II complex exhibited higher selectivity for As(V) over phosphate [59].

Pincus et al. further evaluated transition metal cross-linked chitosan complexes for selective As(V) adsorption over phosphate [86]. Their results showed that Cu(II)-chitosan formed predominantly outer-sphere complexes with As(V), while Fe(III)- and Ni(II)-chitosan formed inner-sphere complexes. Notably, only Fe(III)-chitosan demonstrated true selectivity for both As(III) and As(V) over phosphate, highlighting the importance of metal selection in designing selective adsorbents [86].

#### 3.5.2. Competition Between Cu(II) and Coexisting Species

The adsorption of Cu(II) onto chitosan can be significantly influenced by coexisting species, particularly strong ligands and competing cations.

Strong coordinating ligands represent the most severe interference. Guzman et al. studied Cu(II) sorption by chitosan in the presence of citrate ions [60]. They found that under acidic conditions, Cu(II) forms anionic citrate complexes (Cu(OH)L_2_^−^, CuL^−^), which are captured via electrostatic attraction to protonated amino groups. However, free citrate anions compete for adsorption sites, and significant uptake occurs only when the proportion of anionic copper complexes exceeds that of free ligands [60]. Similarly, EDTA, a common industrial chelating agent, forms highly stable complexes with Cu(II) and can occupy chitosan active sites through its carboxyl groups, substantially suppressing Cu(II) adsorption [87].

Competing metal cations also affect Cu(II) removal. The selectivity order of chitosan for divalent metals is Cu(II) > Ni(II) >> Zn(II) = Co(II) >> Mn(II) [88]. This selectivity is attributed to the favorable five-membered chelate ring formed between Cu(II) and the C2 amino and C3 hydroxyl groups, a configuration less accessible to other metal ions [21]. In binary systems containing both Cu(II) and As(V), An et al. observed that the presence of As(V) led to a slight increase in Cu(II) uptake (from 5.2 to 5.9 μmol/g), while As(V) uptake decreased from 5.6 to 3.6 μmol/g, suggesting that distinct amino group species (NH_2_ or NH_3_^+^) are involved in binding the two contaminants [66].

Solution pH plays a decisive role. At low pH (<4), amino groups are protonated, and H^+^ competes effectively with Cu(II) for coordination sites, resulting in negligible adsorption. Maximum Cu(II) uptake typically occurs at pH 5–7, where deprotonated amino groups form stable chelates without inducing Cu(OH)_2_ precipitation [89]. Background electrolytes such as chloride, nitrate, and sulfate ions generally exhibit minimal interference, as their effects are limited to ionic strength adjustments rather than direct competition [62].

Collectively, the studies reviewed above highlight several aspects of binary system adsorption on chitosan-based materials. First, the selectivity for As(V) over competing anions such as phosphate depends on the coordination geometry of metal cross-linkers. Specifically, in chitosan–copper systems, bidentate Cu(II) complexes favor As(V) chelation, whereas Fe(III)-cross-linked chitosan shows selectivity for both As(III) and As(V) over phosphate [59,86]. Second, evidence from binary As(V)–Cu(II) systems indicates that chitosan can simultaneously remove both contaminants, although competitive interactions reduce As(V) uptake while modestly enhancing Cu(II) adsorption, suggesting distinct binding sites for the two species [66]. Third, strong coordinating ligands such as citrate and EDTA impair the adsorption of Cu(II) more severely than competing divalent cations (e.g., Ni(II), Zn(II), and Cd(II)), which exert only moderate effects due to chitosan’s inherent selectivity for Cu(II) [62,88]. Together, these findings suggest that moving beyond single-ion studies toward multi-component systems is important for the design of chitosan-based adsorbents intended for complex wastewater matrices.

## 4. Conclusions

This review provides a focused assessment of recent advances in chitosan-based adsorbents for the removal of As(V) and Cu(II) from water, with particular emphasis on adsorption mechanisms and the emerging application of electrospun nanofiber technologies. The following conclusions can be drawn from the literature review:

(1) Distinct adsorption behaviors for anions and cations: Chitosan-based adsorbents exhibit different adsorption behaviors for As(V) and Cu(II) due to the pH-sensitive nature of their functional groups. Under acidic conditions, protonated amino groups facilitate the capture of As(V) oxyanions via electrostatic attraction. Under near-neutral conditions, deprotonated amino groups coordinate with adjacent hydroxyl groups to form stable five-membered chelate rings with Cu(II) cations. This duality makes chitosan a versatile material for addressing toxic metal(loid) pollution in composite systems.
(2) Introduction of the “charge adaptation–structure synergy” model: This review presents a conceptual framework that synthesizes current understanding of the distinct adsorption characteristics of chitosan. The model asserts that “charge adaptation” (the pH-dependent protonation state of functional groups) regulates the initial electrostatic attraction, whereas “structure synergy” (the spatial and electronic compatibility between the chitosan backbone and target ions) affects the binding strength and selectivity. For As(V), adsorption primarily relies on charge adaptation, whereas for Cu(II), it predominantly depends on structural synergy. This framework provides a molecular-level basis for the targeted design of chitosan-based adsorbents.
(3) Electrospinning enhances adsorption kinetics: In conventional chitosan adsorbents, active sites are often less accessible, contributing to slower kinetics. In contrast, electrospun chitosan nanofibers offer a high specific surface area, interconnected porosity, and reduced diffusion pathways, which help address this limitation. Recent studies have shown that electrospun chitosan membranes can reduce the time required to reach adsorption equilibrium for arsenic to 0.5–2.6 h [34,36,90], representing a noticeable improvement compared to conventional bead or granule formulations.

Anticipating future developments, the convergence of advanced characterization, computational modeling, and green chemistry principles may facilitate the rational design of next-generation adsorbents. Chitosan-based materials thus represent a promising approach for sustainable water purification and the remediation of toxic metal(loid)s.

## Figures and Tables

**Table 1:** Representative 2025 Review Publications on Electrospun Nanofibers for Water Treatment.

Key Focus	Relevance to This Review	Reference
Synthesis and application studies of chitosan nanofibers, including preparation processes, performance factors, and environmental applications	Focuses specifically on chitosan nanofibers, covering fabrication parameters and environmental applications including toxic metal adsorption, aligning with the core theme of this review.	[38]
Electrospun polyacrylonitrile membranes for water treatment	Discusses electrospun membranes for water treatment, providing general background on nanofiber technology applicable to chitosan-based systems.	[39]
Electrospun nanofibers for pollutant adsorption and separation	Provides comprehensive coverage of adsorption mechanisms in electrospun nanofibers, supporting the mechanistic discussion in Section 3.	[40]
Nanofiber filtration membranes from principles to intelligent applications	Reviews state-of-the-art nanofiber membranes, offering perspective on future directions for chitosan-based filtration systems.	[41]
Electrospinning of chitosan-based nanofibers	Specifically focuses on electrospinning of chitosan nanofibers, covering fabrication innovations and applications, supporting the discussion in Section 3.4.	[42]
Hydrophobic electrospun nanofibers for water treatment: challenges and outlook	Discusses challenges (e.g., stability, scalability) of electrospun nanofibers in water treatment, complementing the future perspective section of this review.	[43]

**Table 2:** Comparison of the mechanisms for the removal of As(V) and Cu(II) from water by chitosan.

Mechanism	As(V), Anion	Cu(II), Cation
Primary Active Site	Protonated amine group (–NH_3_^+^)	Amine group (–NH_2_)
Type of Interaction	Electrostatic Attraction: Interaction between opposite charges.	Coordination/Complexation: Formation of a chelate complex
Reaction Representation	R–NH_3_^+^ + HAsO_4_^2−^ → R–NH_3_^+^HAsO_4_^2−^	R-NH_2_ + Cu(II) → R-NH_2_Cu(II)
Role of pH	Optimal at pH below the pKa of chitosan.	Optimal at pH near or above the pKa of chitosan
Nature of Binding	Outer-sphere complexation (physisorption/ion exchange).	Inner-sphere complexation (chemisorption)

**Table 3:** Comparison of Cu(II) adsorption performance of chitosan-based adsorbents.

Adsorbent System	Qmax (mg/g)	Optimal pH	Equilibrium Time	Reference
*Conventional chitosan-based adsorbents*				
Benzaldehyde-modified chitosan beads	81.8	4.0	~120 min	[61]
Sulfate-modified chitosan hydrogel beads	80.3	5.0	120 min	[75]
Sulfhydryl modified chitosan beads	163.3	5.0	~300 min	[76]
Magnetic chitosan hydrogel beads	-	5.0	24 h	[77]
*Electrospun chitosan-based nanofibers*				
CS/PAN nanofiber membrane	164.3	5.0–6.0	90 min	[78]
PVA/CS/PDA nanofibers	326.5	7.0	~90 min	[79]
Polydopamine-grafted chitosan on porous PLLA nanofibers	270.3	5.0	40 min	[80]

## Data Availability

No new data were created or analyzed in this study. Data sharing is not applicable to this article.

## References

[1] Podgorski J., Berg M. (2020). Global threat of arsenic in groundwater. Science.

[2] Yuan F., Yan D.F., Song S.B., Zhang J.B., Yang Y.Y., Chen Z., Lu J.H., Wang S.M., Sun Y.J. (2025). Removal of heavy metals from water by adsorption on metal organic frameworks: Research progress and mechanistic analysis in the last decade. Chem. Eng. J..

[3] Sadee B.A., Zebari S.M.S., Galali Y., Saleem M.F. (2025). A review on arsenic contamination in drinking water: Sources, health impacts, and remediation approaches. RSC Adv..

[4] Al-Saydeh S.A., El-Naas M.H., Zaidi S.J. (2017). Copper removal from industrial wastewater: A comprehensive review. J. Ind. Eng. Chem..

[5] Chigome S., Andala D., Kabomo M., Mobegi E. (2022). Perspectives on the development of filter media for point of use water filters: Case study of arsenate removal. Front. Chem..

[6] de Conti A., Madia F., Schubauer-Berigan M.K., Benbrahim-Tallaa L. (2025). Carcinogenicity of some metals evaluated by the IARC Monographs: A synopsis of the evaluations of arsenic, cadmium, cobalt, and antimony. Toxicol. Appl. Pharmacol..

[7] Ling W., Li S., Zhu Y., Wang X., Jiang D., Kang B. (2025). Inducers of autophagy and cell death: Focus on copper metabolism. Ecotoxicol. Environ. Saf..

[8] Binesh A., Venkatachalam K. (2024). Copper in human health and disease: A comprehensive review. J. Biochem. Mol. Toxicol..

[9] Malhotra N., Ger T.-R., Uapipatanakul B., Huang J.-C., Chen K.H.-C., Hsiao C.-D. (2020). Review of copper and copper nanoparticle toxicity in fish. Nanomaterials.

[10] Taylor A.A., Tsuji J.S., Garry M.R., McArdle M.E., Goodfello W.L., Adams W.J., Menzie C.A. (2020). Critical review of exposure and effects: Implications for setting regulatory health criteria for ingested copper. Environ. Manag..

[11] Ni S., Liu G., Zhao Y., Zhang C., Wang A. (2023). Distribution and source apportionment of heavy metals in soil around Dexing copper mine in Jiangxi Province, China. Sustainability.

[12] Wei C., Wang C., Yang L. (2009). Characterizing spatial distribution and sources of heavy metals in the soils from mining-smelting activities in Shuikoushan, Hunan Province, China. J. Environ. Sci..

[13] Huang W., Liu Y., Bi X., Wang Y., Li H., Qin J., Chen J., Ruan Z., Chen G., Qiu R. (2025). Source-specific soil heavy metal risk assessment in arsenic waste mine site of Yunnan: Integrating environmental and biological factors. J. Hazard. Mater..

[14] Gahrouei A.E., Rezapour A., Pirooz M., Pourebrahimi S. (2024). From classic to cutting-edge solutions: A comprehensive review of materials and methods for heavy metal removal from water environments. Desalination Water Treat..

[15] Barakat M.A. (2011). New trends in removing heavy metals from industrial wastewater. Arab. J. Chem..

[16] Crini G., Lichtfouse E. (2019). Advantages and disadvantages of techniques used for wastewater treatment. Environ. Chem. Lett..

[17] Kaur S., Dhillon G.S. (2014). The versatile biopolymer chitosan: Potential sources, evaluation of extraction methods and applications. Crit. Rev. Microbiol..

[18] Hegde S., Selvaraj S. (2024). Chitosan: An in-depth analysis of its extraction, applications, constraints, and future prospects. J. Microbiol. Biotechnol. Food Sci..

[19] Vakili M., Rafatullah M., Salamatinia B., Abdullah A.Z., Ibrahim M.H., Tan K.B., Gholami Z., Amouzgar P. (2014). Application of chitosan and its derivatives as adsorbents for dye removal from water and wastewater: A review. Carbohydr. Polym..

[20] Kaczorowska M.A., Bozejewicz D. (2024). The Application of chitosan-based adsorbents for the removal of hazardous pollutants from aqueous solutions—A review. Sustainability.

[21] Guibal E. (2004). Interactions of metal ions with chitosan-based sorbents: A review. Sep. Purif. Technol..

[22] Patel P.K., Uppaluri R.V. (2025). Chitosan and its functionalized derivatives for heavy metal ion elimination: A review of synthesis, mechanisms, and characterization studies. Adv. Colloid Interface Sci..

[23] Periyannan K., Selvaraj H., Subbu B., Pallikondaperumal M., Karuppiah P., Rajabathar J.R., Al-Lohedan H., Thangarasu S. (2023). Green fabrication of chitosan from marine crustaceans and mushroom waste: Toward sustainable resource utilization. Green Process. Synth..

[24] Yadav M., Goswami P., Paritosh K., Kumar M., Pareek N., Vivekanand V. (2019). Seafood waste: A source for preparation of commercially employable chitin/chitosan materials. Bioresour. Bioprocess..

[25] Pakizeh M., Moradi A., Ghassemi T. (2021). Chemical extraction and modification of chitin and chitosan from shrimp shells. Eur. Polym. J..

[26] Rødde R.H., Einbu A., Vårum K.M. (2008). A seasonal study of the chemical composition and chitin quality of shrimp shells obtained from northern shrimp (*Pandalus borealis*). Carbohydr. Polym..

[27] Hellmann M.J., Marongiu G.L., Gorzelanny C., Moerschbacher B.M., Cord-Landwehr S. (2025). Hydrolysis of chitin and chitosans by the human chitinolytic enzymes: Chitotriosidase, acidic mammalian chitinase, and lysozyme. Int. J. Biol. Macromol..

[28] Nordtveit R.J., Vårum K.M., Smidsrød O. (1994). Degradation of fully water-soluble, partially N-acetylated chitosans with lysozyme. Carbohydr. Polym..

[29] Koumentakou I., Meretoudi A., Emmanouil C., Kyzas G.Z. (2025). Environmental toxicity and biodegradation of chitosan derivatives: A comprehensive review. J. Ind. Eng. Chem..

[30] Varma A.J., Deshpande S.V., Kennedy J.F. (2004). Metal complexation by chitosan and its derivatives: A review. Carbohydr. Polym..

[31] Roy B.K., Tahmid I., Rashid T.U. (2021). Chitosan-based materials for supercapacitor applications: A review. J. Mater. Chem. A.

[32] Doobi F.A., Mir F.Q. (2024). Exploring the development of natural biopolymer (chitosan)-based proton exchange membranes for fuel cells: A review. Results Surf. Interfaces.

[33] Alatawi R.A.S. (2025). Electrospun nanofiber chitosan/polyvinyl alcohol loaded with metal organic framework nanofiber for efficient adsorption and removal of industrial dyes from waste water: Adsorption isotherm, kinetic, thermodynamic, and optimization via Box-Behnken design. Int. J. Biol. Macromol..

[34] Min L., Yuan Z., Zhong L., Liu Q., Wu R., Zheng Y. (2015). Preparation of chitosan based electrospun nanofiber membrane and its adsorptive removal of arsenate from aqueous solution. Chem. Eng. J..

[35] Min L., Yang L., Wu R., Zhong L., Yuan Z., Zheng Y. (2019). Enhanced adsorption of arsenite from aqueous solution by an iron-doped electrospun chitosan nanofiber mat: Preparation, characterization and performance. J. Colloid Interface Sci..

[36] Min L., Ma Y., Zhang B., He D., Chen J., Li X., Wang S., Chi Y. (2024). Electrospinning chitosan/Fe-Mn nanofibrous composite for efficient and rapid removal of arsenite from water. Toxics.

[37] Wang Z., Kang S.B., Yun H.J., Won S.W. (2023). Efficient removal of arsenate from water using electrospun polyethylenimine/polyvinyl chloride nanofiber sheets. React. Funct. Polym..

[38] Zhong L., Yang J., Zhang T., Wu L., Wu Y., Zhang J., Li M., Wu K., Shang M., Liu C. (2025). Synthesis and application studies of chitosan nanofibers. Int. J. Biol. Macromol..

[39] Shakiba M., Faraji M., Jouybar S., Foroozandeh A., Bigham A., Abdouss M., Saidi M., Vatanpour V., Varma R.S. (2025). Advanced nanofibers for water treatment: Unveiling the potential of electrospun polyacrylonitrile membranes. Environ. Res..

[40] Majlesi M., Assadpour E., Tavassoli M., Jafari S.M. (2025). Electrospun nanofibers for the adsorption and separation of pollutants from wastewater: New opportunities and recent advances. Adv. Colloid Interface Sci..

[41] Shi S., Bai W., Chen X., Si Y., Zhi C., Wu H., Su Y., Cai W., Fei B., Kan C.W. (2025). Advances in nanofiber filtration membranes: From principles to intelligent applications. Adv. Funct. Mater..

[42] Li S., Li Y., Zhang X., Jiang D., Kong L. (2025). Electrospinning of chitosan-based nanofibers: Innovations in fabrication and applications. J. Agric. Food Res..

[43] Janjhi F.A., Chandio I., Janwery D., Vatanpour V., Castro-Muñoz R. (2025). A review on hydrophobic electrospun nanofibers-based materials and membranes for water treatment: Challenges, outlook, and stability. Sep. Purif. Technol..

[44] Strand S.P., Tømmeraas K., Vårum K.M., Østgaard K. (2001). Electrophoretic light scattering studies of chitosans with different degrees of N-acetylation. Biomacromolecules.

[45] Gupta D., Haile A. (2007). Multifunctional properties of cotton fabric treated with chitosan and carboxymethyl chitosan. Carbohydr. Polym..

[46] Gupta A., Chauhan V.S., Sankararamakrishnan N. (2009). Preparation and evaluation of iron–chitosan composites for removal of As(III) and As(V) from arsenic contaminated real life groundwater. Water Res..

[47] Wan M.-W., Kan C.-C., Rogel B.D., Dalida M.L.P. (2010). Adsorption of copper (II) and lead (II) ions from aqueous solution on chitosan-coated sand. Carbohydr. Polym..

[48] Schatz C., Viton C., Delair T., Pichot C., Domard A. (2003). Typical physicochemical behaviors of chitosan in aqueous solution. Biomacromolecules.

[49] Lopez-Leon T., Carvalho E.L.S., Seijo B., Ortega-Vinuesa J.L., Bastos-González D. (2005). Physicochemical characterization of chitosan nanoparticles: Electrokinetic and stability behavior. J. Colloid Interface Sci..

[50] Franca E., Lins R., Freitas L., Straatsma T. (2008). Characterization of Chitin and Chitosan Molecular Structure in Aqueous Solution. J. Chem. Theory Comput..

[51] Smith E., Naidu R., Alston A.M. (1998). Arsenic in the Soil Environment: A Review. Adv. Agron..

[52] Zeng H., Ma G., Zheng X., Lin D., Zhang J., Li D. (2025). Preparation, application and regeneration of chitosan composite adsorbents for arsenic removal: A review from a sustainable perspective. Int. J. Biol. Macromol..

[53] Dambies L., Vincent T., Guibal E. (2002). Treatment of arsenic-containing solutions using chitosan derivatives: Uptake mechanism and sorption performances. Water Res..

[54] Dambies L., Guibal E., Roze A. (2000). Arsenic(V) sorption on molybdate-impregnated chitosan beads. Colloids Surf. A.

[55] Sun S., Zeng H., Xu H., Zhao W., Qi W., Hao R., Zhang J., Li D. (2023). Adsorption of As (V) and P (V) by magnetic iron-based alginate-chitosan beads: Competitive adsorption and reduction mechanism of As (V) induced by Fe (II). Colloids Surf. A.

[56] He W., Mallavarapu M., Naidu R. (2009). Toxicity of tri- and penta-valent arsenic, alone and in combination, to the cladoceran Daphnia carinata: The influence of microbial transformation in natural waters. Environ. Geochem. Health.

[57] Hudek M., Johnston K., Kubiak-Ossowska K., Ferro V.A., Mulheran P.A. (2024). Molecular dynamics study of chitosan adsorption at a silica surface. J. Phys. Chem. C.

[58] Meza-González B., Jacinto M.M., Brito-Flores L., Cortes-Guzman F., Gómez-Espinosa R.M. (2024). Interaction between chitosan and arsenic acid. Chem. Phys..

[59] Yamani J.S., Lounsbury A.W., Zimmerman J.B. (2016). Towards a selective adsorbent for arsenate and selenite in the presence of phosphate: Assessment of adsorption efficiency, mechanism, and binary separation factors of the chitosan-copper complex. Water Res..

[60] Rhazi M., Desbrières J., Tolaimate A., Rinaudo M., Vottero P., Alagui A. (2002). Contribution to the study of the complexation of copper by chitosan and oligomers. Polymer.

[61] Law S.C., Wan Ngah W.S., Hanafiah M.A.K.M., Perumal V., Balan T., Mohideen M., Sandanamsamy S.S., Selvarajoo P.D., Fatinathan S. (2025). Kinetic, isotherm, and mechanistic insights into Cu^2+^ and Ni^2+^ ions removal using benzaldehyde–modified chitosan beads in batch adsorption and fixed-bed column systems. Pure Appl. Chem..

[62] Guzman J., Saucedo I., Revilla J., Navarro R., Guibal E. (2003). Copper sorption by chitosan in the presence of citrate ions: Influence of metal speciation on sorption mechanism and uptake capacities. Int. J. Biol. Macromol..

[63] Li H.-F., Mu Y.-F., Zhang J., Lan D., Guo X.-M., Tang J., Wang Y., Wang Y.-Y. (2025). Chitosan tailored SiO_2_ aerogel for efficient removal of copper ions. Int. J. Biol. Macromol..

[64] Wu Z., Su J., Cong X., Zhao D., Wu J., Zhou H., Huang M., Zheng J., Zhao D., Sun B. (2024). Preparation, characterization, and mechanism of swelling and Cu^2+^ adsorption by alginate-based beads enhanced with Huangshui polysaccharides. J. Clean. Prod..

[65] Chen B., Chen Y., Cao Y., Huang J., Chen X., Pan X. (2024). Collaboratively scavenge tetracycline and Cu^2+^ from their combined system by Fe^3+^-modified magnetic chitosan: Performance, mechanisms, and dynamic sorption process. Chem. Eng. J..

[66] An B. (2020). Cu(II) and As(V) adsorption kinetic characteristic of the multifunctional amino groups in chitosan. Processes.

[67] Joseph T., Jacob M., Nair V., Varkey J. (2021). Removal of metal ions using Chitosan based electro spun nanofibers: A review. Nanosyst. Phys. Chem. Math..

[68] Naseri N., Algan C., Jacobs V., John M., Oksman K., Mathew A.P. (2014). Electrospun chitosan-based nanocomposite mats reinforced with chitin nanocrystals for wound dressing. Carbohydr. Polym..

[69] Min L.L., Zhong L.B., Zheng Y.M., Liu Q., Yuan Z.H., Yang L.M. (2016). Functionalized chitosan electrospun nanofiber for effective removal of trace arsenate from water. Sci. Rep..

[70] Ramakrishna S., Fujihara K., Teo W.-E., Yong T., Ma Z., Ramaseshan R. (2006). Electrospun nanofibers: Solving global issues. Mater. Today.

[71] Crini G. (2005). Recent developments in polysaccharide-based materials used as adsorbents in wastewater treatment. Prog. Polym. Sci..

[72] Yang D., Li L., Chen B., Shi S., Nie J., Ma G. (2019). Functionalized chitosan electrospun nanofiber membranes for heavy-metal removal. Polymer.

[73] Elamin N.Y., Elamin M.R., Alhussain H., Alotaibi M.T., Alluhaybi A.A., El-Bindary A.A. (2025). Electrospun nanofibers of polycaprolactone loaded with chitosan/carbon quantum dots composite for adsorptive removal of Cd(II) ions from wastewater: Adsorption, kinetics, thermodynamics, and BOX Behnken design. Int. J. Biol. Macromol..

[74] Zhang K., Zhu C., Xie L., Zhang L., Chai X., Wu C., Wang S., Peng W., Du G., Xu K. (2025). Facile fabrication of electrospun hybrid nanofibers integrated cellulose, chitosan with ZIF-8 for efficient remediation of copper ions. Carbohydr. Polym..

[75] Chatterjee S., Guha A.K., Chatterjee B., Banerjee P., Singha N.R. (2025). Sulphate-modified chitosan hydrogel beads a recyclable bio-adsorbent for Cu(II): Experimental and theoretical studies toward nitrogen-/oxygen-donor selective binding and 1:1 complexation. Int. J. Biol. Macromol..

[76] Yang Y., Zeng L., Lin Z., Jiang H., Zhang A. (2021). Adsorption of Pb^2+^, Cu^2+^ and Cd^2+^ by sulfhydryl modified chitosan beads. Carbohydr. Polym..

[77] Afifah Khalil N., Syafiqah Abdul Rahman A., Mubin Abu Huraira A., Nur Dalillah Fattin Janurin S., Noor Syimir Fizal A., Ahmad N., Zulkifli M., Sohrab Hossain M., Naim Ahmad Yahaya A. (2023). Magnetic chitosan hydrogel beads as adsorbent for copper removal from aqueous solution. Mater. Today Proc..

[78] Zhang H., Yao C., Qin X. (2021). A visually observable copper ion adsorption membrane by electrospinning combined with copper ion probe. Fibers Polym..

[79] Li C., Fu L., Deng S., Wang H., Jia L. (2024). Polydopamine-functionalized electrospun poly(vinyl alcohol)/chitosan nanofibers for the removal and determination of Cu(II). Int. J. Biol. Macromol..

[80] Zia Q., Tabassum M., Meng J., Xin Z., Gong H., Li J. (2021). Polydopamine-assisted grafting of chitosan on porous poly (L-lactic acid) electrospun membranes for adsorption of heavy metal ions. Int. J. Biol. Macromol..

[81] Chen C.C., Chung Y.C. (2006). Arsenic removal using a biopolymer chitosan sorbent. J. Environ. Sci. Health Part A Toxic/Hazard. Subst. Environ. Eng..

[82] Gérente C., Andrès Y., McKay G., Le Cloirec P. (2010). Removal of arsenic(V) onto chitosan: From sorption mechanism explanation to dynamic water treatment process. Chem. Eng. J..

[83] de Souza J.R., Sato T.P., Borges A.L.S. (2017). Scaffold architecture for dental biomaterials: Influence of process parameters on the structural morphology of chitosan electrospun fibers. Braz. Dent. Sci. J..

[84] Abdelhady S., El-Desouky A.R., Kassab A.M.F., Barakat W., Zoalfakar S. (2023). Optimization of electrospun chitosan/polyethylene oxide hybrid nanofibril composite via response surface methodology. J. Thermoplast. Compos. Mater..

[85] Rendon-Heredia O., Vasquez-Garcia S.R., Flores-Ramirez N., Fernandez-Quiroz D., Sanchez-Orozco R. (2024). Chitosan films: Tailoring properties for sustainable applications through casting and electrospinning. MRS Adv..

[86] Pincus L.N., Petrovic P.V., Gonzalez I.S., Stavitski E., Fishman Z.S., Rudel H.E., Anastas P.T., Zimmerman J.B. (2021). Selective adsorption of arsenic over phosphate by transition metal cross-linked chitosan. Chem. Eng. J..

[87] Gylienė O., Nivinskienė O., Razmutė I. (2006). Copper (II)–EDTA sorption onto chitosan and its regeneration applying electrolysis. J. Hazard. Mater..

[88] Inoue K., Yoshizuka K., Ohto K. (1999). Adsorptive separation of some metal ions by complexing agent types of chemically modified chitosan. Anal. Chim. Acta.

[89] Verbych S., Bryk M., Chornokur G., Fuhr B. (2005). Removal of copper (II) from aqueous solutions by chitosan adsorption. Sep. Sci. Technol..

[90] Horzum N., Demir M.M., Nairat M., Shahwan T. (2013). Chitosan fiber-supported zero-valent iron nanoparticles as a novel sorbent for sequestration of inorganic arsenic. RSC Adv..

